# NOVEL MOLECULAR MECHANISMS UNDERLYING THE ASSOCIATION BETWEEN PLCG2, ALZHEIMER’S DISEASE, AND LONGEVITY

**DOI:** 10.1093/geroni/igad104.0326

**Published:** 2023-12-21

**Authors:** Juan Palavicini, Juliet Garcia Rogers, Henry Miller, Susan Weintraub, Sarah Hopp

**Affiliations:** The Barshop Institute for Longevity and Aging Studies, San Antonio, Texas, United States; UT Health SA, San Antonio, Texas, United States; Bioinformatics Research Network, Atlanta, Georgia, United States; UT Health SA, San Antonio, Texas, United States; UT Health SA, San Antonio, Texas, United States

## Abstract

Phospholipase C-gamma-2 (PLCγ2) catalyzes the hydrolysis of the membrane phosphatidylinositol-4,5-bisphosphate (PIP2) to form diacylglycerol (DAG) and inositol trisphosphate (IP3), which subsequently feed into numerous downstream signaling pathways. PLCG2 rare coding variants are associated with both reduced (P522R) and increased (M28L) risk of Alzheimer’s disease (AD) and with longevity (P522R). In the brain, PLCG2 is primarily expressed by microglia, where it is proposed to regulate phagocytosis, secretion of cytokines and chemokines, cell survival and proliferation. We analyzed the brains of 3-month-old PLCγ2 knockout (KO), heterozygous (HET), and wild-type (WT) mice using multi-omic and histological approaches. Lipidomic analyses revealed sex-specific changes in total brain PIP2 and DAG content in KOs. Surprisingly, we found that PLCγ2 depletion led to significant decreases in myelin-enriched lipids including cerebrosides, sulfatides and phosphatidylethanolamine plasmalogens. Consistent with our lipidomics results, gene expression profiling revealed sex-specific changes in myelin-related genes. Further, in line with the available literature, gene expression profiling revealed PLCγ2 depletion led to subtle changes on microglia phenotype, suggestive of reduced microglia reactivity. Immunofluorescence confirmed subtle differences in density of microglia and oligodendrocytes in KOs. Exploratory proteomic pathway analyses revealed changes in KO and HET females compared to WTs, with over-abundant proteins pointing to mTOR signaling, and under-abundant proteins pointing to oligodendrocytes. Furthermore, a PLCG2 M28L knockin mouse model displayed phenotypes of accelerated aging, including exacerbated thymic atrophy, increased frailty scores, and impaired spatial memory. In summary, our study unraveled novel putative mechanisms underlying the association between PLCγ2, AD, and longevity (mTOR signaling and myelin homeostasis).

